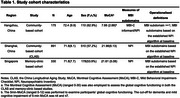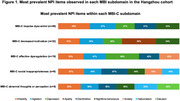# Impulse dyscontrol as the leading mild behavioral impairment domain among Chinese dementia‐free older adults

**DOI:** 10.1002/alz70857_101480

**Published:** 2025-12-25

**Authors:** Yingqi Liao, Yaping Zhang, Eric Tan, Christopher Chen, Xin Xu

**Affiliations:** ^1^ Memory, Ageing, and Cognition Centre (MACC), Department of Pharmacology, Yong Loo Lin School of Medicine, National University of Singapore, Singapore, Singapore; ^2^ School of Public Health, the Second Affiliated Hospital of School of Medicine, Zhejiang University, Hangzhou, Zhejiang, China; ^3^ Department of Psychological Medicine, Yong Loo Lin School of Medicine, National University of Singapore, Singapore, Singapore, Singapore

## Abstract

**Background:**

Life‐life emergent and persistent NPS, defined as mild behavioral impairment (MBI), is considered as an early marker for cognitive decline and dementia conversion. However, clinical presentation of MBI remained less studied among older adults of Chinese ethnicity. This study aims to examine the leading MBI domain in Chinese dementia‐free older adults, as well as to evaluate the optimal use of Neuropsychiatric Inventory (NPI) in capturing MBI subdomains identified by Mild Behavioral Impairment‐Checklist (MBI‐C).

**Method:**

Informant‐based NPI data of three original datasets, including two community‐dwelling cohorts (Hangzhou community cohort and the China Longitudinal Aging Study) and one memory‐clinic based cohort in Singapore, were transformed based on the established algorithm for MBI subdomains and pooled using the random‐effect meta‐analysis method. Using the Hangzhou cohort, we further examined the frequency of NPI items in individual MBI subdomains (sum scores of MBI‐C subdomain ≥ 1). To understand the overlapping NPS in the leading MBI subdomain, the principal factor analysis (PCA) with Promax rotation on NPI was further conducted. The number of factors for extraction was determined by the visual inspection of the scree plots, as well the number of eigenvalues ≥ 1.

**Result:**

A total of 1,396 dementia‐free participants aged ≥ 50 years old were included in the meta‐analyses (Table 1). The impulse dyscontrol domain (11%, 95%CI=0.004–0.31) was the most prevalent across cohorts, while the social inappropriateness (2%, 95%CI=0–0.11) was the least observed domain. In the Hangzhou cohort, 28% of participants exhibited impulse dyscontrol measured by MBI‐C, with the most common NPI items being irritability (45%) and agitation (37%) (Figure 1). PCA analysis further revealed three extracted factors of NPI items (factor loading cutoff > 0.4), with agitation, disinhibition, irritability, delusion and hallucination loaded on factor 1, depression and anxiety loaded on factor 2 and elation loaded onto its own factor 3.

**Conclusion:**

The leading MBI subdomain among Chinese older adults is impulse dyscontrol, which was different from affective dysregulation observed in western populations. Further studies are encouraged to examine the presentation and mechanisms of the impulse dyscontrol domain in Chinese populations, which could facilitate the development of specific and effective treatment.